# One-Pot Synthesis of Charged Amphiphilic Diblock and Triblock Copolymers Via High-Throughput Cu(0)-Mediated Polymerization

**DOI:** 10.3390/polym9080320

**Published:** 2017-07-30

**Authors:** Lenny Voorhaar, Richard Hoogenboom

**Affiliations:** 1 Supramolecular Chemistry Group, Department of Organic and Macromolecular Chemistry, Ghent University, Krijgslaan 281 S4, 9000 Ghent, Belgium; lenny.voorhaar@gmail.com; 2 SIM vzw, Technologiepark 935, 9052 Zwijnaarde, Belgium

**Keywords:** controlled radical polymerization, high-throughput polymerization, poly(acrylate)s

## Abstract

Block copolymers containing functionalized monomers, for example those containing charged groups, can be used for many purposes, one of which is the design of polymeric supramolecular materials based on electrostatic interactions. In this paper the synthesis of diblock copolymers and ABA-triblock copolymers containing poly(*n*-butyl acrylate) as a first or middle block and poly(2-(dimethylamino)ethyl acrylate), poly(1-ethoxyethyl acrylate) and poly(1-ethoxyethyl-2-carboxyethyl acrylate) as second or outer blocks, resulting in block copolymers that can contain positive or negative charges, is reported. The polymerizations were performed and optimized via one-pot sequential monomer addition reactions via Cu(0)-mediated polymerization using an automated parallel synthesizer. Different initiators, monomer concentrations and polymerization times were tested. While a bromide-containing initiator led to the best results for most monomers, when polymerizing 2-(dimethylamino)ethyl acrylate the use of a chloride-containing initiator was necessary. Due to the slower polymerization using this initiator, a longer polymerization time was needed before addition of the second monomer. Using the optimized conditions, the diblock and triblock copolymers could be synthesized with good control over molecular weight and dispersities around 1.1 were obtained.

## 1. Introduction

Recently, we reported the optimization of the Cu(0)-mediated polymerization of *n*-butyl acrylate and 2-methoxyethyl acrylate using an automated parallel synthesizer [1]. We also showed that, under optimized conditions, diblock copolymers could be synthesized by addition of a second monomer to the reaction mixture after most of the first monomer was polymerized. Here we expand this work by optimizing the one-pot automated synthesis of amphiphilic diblock and triblock copolymers using different, more challenging monomers. We have recently shown that these types of amphiphilic triblock copolymers can be used to form novel supramolecular materials with interesting properties, such as self-healing and a rubber plateau that extends beyond the glass transition temperature of the hard phase [2].

Cu(0)-mediated polymerization is very well suited for one-pot block copolymerizations based on the excellent control over the polymerization [3]. An earlier pioneering paper showed the synthesis of multiblock copolymers containing very short blocks of monomers that were added sequentially to the reaction [4], while other more recent publications also reported the synthesis of multiblock copolymers with longer blocks [5,6,7,8]. Synthesis of star-shaped block copolymers was also reported [9] and the synthesis of multiblock polyacrylamides was also shown recently [10,11]. Generally, almost full conversion of each block was obtained and dispersions were low even after the addition of several blocks, although some termination of chains can often be observed when the number of blocks increases. In each of these examples, relatively simple acrylates without functional groups were used, such as methyl acrylate and butyl acrylate. Moreover, each monomer had to be added manually, requiring labor-intensive work and careful planning of experiments. Here we aim to synthesize amphiphilic block copolymers containing functional acrylates using Cu(0)-mediated polymerization with sequential monomer addition in an automated system. This system is ideal for these reactions, as liquids can be transferred at precisely set time intervals allowing continuous monitoring of the polymerization kinetics (also throughout the night), even if the reactions take several days.

*n*-Butyl acrylate (BA) is used as a hydrophobic first or middle block in these polymerizations. The first hydrophilic block that will be attached is poly(2-(dimethylamino)ethyl acrylate) (PDMAEA), an amine-functionalized monomer that can carry a positive charge when protonated. The amine may create problems during Cu(0)-mediated polymerization because of its ability to act as a ligand for copper ions or by reaction with the halogen end group of the initiator or growing polymer chain [12]. A similar type of quarternization reaction was reported earlier for Cu(0)-mediated polymerization with Me_6_TREN as ligand that was quarternized with EBP and bromine-containing chain ends [13]. However, Me_6_TREN is usually present in much lower amounts than the monomer, so these reactions may be much more pronounced when using an amine-functionalized monomer. Recent reports of Cu(0)-mediated polymerization of DMAEA also showed limitations such as termination and side reactions [14,15].

**Figure 1:**
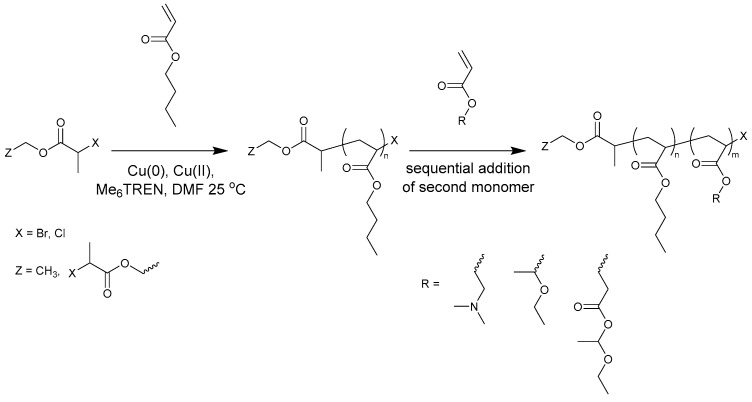
Synthesis of block copolymers by sequential monomer addition.

A second hydrophilic block will be incorporated of 1-ethoxyethyl acrylate (EEA), which is readily deprotected to form acrylic acid (AA) when it is heated or treated with water [16,17,18]. The thermal dissociation is reported to take place at 140 °C in a thin film [16], but can also slowly proceed at lower temperatures [17,19]. This is an alternative to the often-used *tert*-butyl acrylate, which can be converted into AA via acidic hydrolysis [20,21]. EEA was chosen over *tert*-butyl acrylate as a mild thermal treatment for deprotection was preferred over additional hydrolysis reactions. PAA is a water-soluble polymer that carries a negative charge when deprotonated. However, free AA cannot be present during ATRP or Cu(0)-mediated polymerization due to its disruptive interaction with the catalytic system by strong coordination of the copper ions and protonation of the nitrogen-containing ligands. A related newly developed monomer that will also be used to prepare the hydrophilic blocks is ethoxyethyl-protected 2-carboxyethyl acrylate (proCEA), which can be deprotected to 2-carboxyethyl acrylate (CEA). Both monofunctional and bifunctional initiators were used for the sequential Cu(0)-mediated polymerizations to be able to synthesize diblock and triblock copolymers (Figure 1). The amphiphilic ABA-triblock copolymers were also manually synthesized on a larger scale.

## 2. Materials and Methods 

### 2.1. Materials

*N,N*-Dimethylformamide (DMF, dry) and tetrahydrofuran (THF, dry, unstabilized and free of peroxides) were obtained from a solvent purification system (Meyer, Vallejo, CA, USA, custom made with a nitrogen, aluminum oxide drying system). Copper(II)bromide (CuBr_2_, 99%) was purchased from Fluka (Buchs, Switzerland). Dichloromethane (99.8%), ethyl 2-bromopropionate (EBP, 99%), ethyl 2-chloropropionate (ECP, 97%), copper(II)chloride (CuCl_2_, 97%), acrylic acid (99%), ethyl vinyl ether (99%), phosphoric acid (99.99%), 4-(dimethylamino)pyridine (99%), ethylene glycol (99%), 2-chloropropionic acid (92%), 2-bromopropionyl bromide (97%), pyridine (99.8%), sodium bicarbonate (NaHCO_3_, 99.7%), tris(2-aminoethyl)amine (96%), formic acid (95%), formaldehyde (37% solution) and inhibitor removers were purchased from Sigma-Aldrich (St. Louis, MI, USA). *N*-(3-Dimethylaminopropyl)-*N*'-ethylcarbodiimide hydrochloride was purchased from Iris Biotech (Marktredwitz, Germany). Hydrochloric acid (HCl, 37% solution) and sodium chloride (99%) were purchased from Acros (Geel, Belgium). Magnesium sulfate (MgSO_4_, dried) was purchased from Fisher Scientific (Hampton, NH, USA). Aluminum oxide (90 standardized) was purchased from Merck (Kenilworth, NJ, USA). CupriSorb was purchased from Amazon (Seattle, WA, USA). All were used as received. *N*-butyl acrylate (BA, 99%) was purchased from Sigma-Aldrich and purified by passing over a basic aluminum oxide column to remove the inhibitor. 2-(Dimethylamino)ethyl acrylate (DMAEA, 97%) was purchased from TCI (Zwijndrecht, Belgium) and the inhibitor was removed by stirring with inhibitor remover that was removed by filtration. Tris[2-(dimethylamino)ethyl]amine (Me_6_TREN) was synthesized according to a previously published procedure [22]. Pre-cut copper wire (Sigma-Aldrich, 99.9%) was stirred in sulfuric acid, milliQ water and acetone before use.

### 2.2. Automated Cu(0)-Mediated Polymerization

Reactions were performed using a Chemspeed ASW2000 automated synthesizer (Füllinsdorf, Switzerland) equipped with 16 parallel reactors of 13 mL, a Huber Petite Fleur thermostat (Offenburg, Germany) for heating/cooling, a Huber Ministat 125 for reflux and a Vacuubrand PC 3000 vacuum pump. Polymerization of BA was performed using the optimized reaction conditions from our previous paper [1], which are a [BA]:[I]:[Me_6_TREN]:[Cu(II)] ratio of 50:1:0.15:0.1 at 3.0 M in DMF at 25 °C and 12.5 mm^2^/mL Cu(0) wire, with a reaction volume of 4 mL. A solution of the second monomer was added to the reactors after a certain time for the synthesis of block copolymers. All components were dissolved in DMF and degassed via bubbling with argon for at least 30 min before these stock solutions were introduced into the robot system and then kept under argon atmosphere. The reactors were degassed through ten vacuum-argon cycles, after which they were continuously kept under argon overpressure. The hood of the automated synthesizer was continuously flushed with nitrogen (10 L/min) to ensure an inert atmosphere. Using the syringe of the automated synthesizer, the stock solutions were transferred to the reactors. At preset time intervals during the reactions, 50 μL samples were taken and directly injected into 1.5 mL sample vials, each containing a 0.1 mg/mL solution of phenothiazine in ~1.5 mL THF to quench the reactions.

### 2.3. Gas Chromatography (GC)

Samples were measured with GC to determine the monomer conversion from the ratio of the integrals from the monomer and the internal standard. GC was performed on an Agilent 7890A system (Santa Clara, CA, USA), which was equipped with a VWR Carrier-160 hydrogen generator (Radnor, PA, USA), an FID detector and an Agilent HP-5 column of 30 m length and 0.320 mm diameter. The inlet was set to 250 °C with a split injection ratio of 25:1. The carrier gas was hydrogen at a flow rate of 2 mL/min. The temperature program used was injection at 50 °C, after which the temperature was increased by 20 °C/min from 50 to 120 °C, followed by a ramp of 50 °C/min to 300 °C. 

### 2.4. Size Exclusion Chromatography (SEC)

Residual copper was removed from the samples on a short aluminum oxide column before the SEC measurements. SEC was performed on a Varian PL-GPC 50 Plus system (Palo Alto, CA, USA) equipped with two PLgel 5 µm MIXED-D columns, a PL-AS RT autosampler and five detectors: RI, light scattering at 15° and 90°, a viscometer and a UV Knauer Wellchrom Spectro-Photometer K-2501 (Berlin, Germany). THF was used as the eluent with a flow of 1 mL/min. Molecular weights were determined based on the RI signal and calculated using polystyrene standards.

### 2.5. Synthesis of Ethylene Glycol Bis(2-bromopropionyl) Ethane (BPE)

BPE was synthesized following a previously published method [23].

### 2.6. Synthesis of Ethylene Glycol Bis(2-chloropropionyl) Ethane (CPE)

A solution of ethylene glycol (2.00 g, 32.2 mmol) and 2-chloropropionic acid (8.39 g, 77.3 mmol) in dichloromethane (30 mL) was cooled in an ice bath and a solution of *N*-(3-dimethylaminopropyl)-*N*‘-ethylcarbodiimide hydrochloride (16.57 g, 80.6 mmol) and 4-(dimethylamino)pyridine (0.79 g, 6.4 mmol) in dichloromethane (60 mL) was added dropwise. The reaction was allowed to warm up to room temperature and stirred overnight. It was then washed with 1 M HCl, distilled water, a NaHCO_3_ solution and brine. The solution was dried with MgSO_4_, filtered and the solvent was removed under reduced pressure to yield 7.35 g (94%) of CPE as a light yellow liquid.

^1^H NMR (CDCl_3_, 300 MHz) δ: 4.42 ppm (4 H, s, 2 C*H*_2_–CH_2_), 4.41 ppm (2 H, q, 2 C*H*–CH_3_), 1.70 ppm (6 H, d, 2 CH–C*H*_3_) 

### 2.7. Synthesis of 1-Ethoxyethyl Acrylate (EEA) and Protected 2-Carboxyethyl Acrylate (ProCEA) 

EEA was synthesized following a previously published procedure and distilled prior to use. ProCEA was made in a very similar procedure, also see the Supplementary Information (Experimental section) [16]. 

^1^H NMR EEA (CDCl_3_, 300 MHz) δ: 6.40 ppm (1 H, dd, C*H*_2_=CH–C(O)), 6.10 ppm (1 H, dd, CH_2_=C*H*–C(O)), 5.99 ppm (1 H, q, CH_3_–C*H*–(O)_2_), 5.82 ppm (1 H, dd, C*H*_2_=CH–C(O)), 3.70 ppm (1 H, m, CH_3_–C*H*_2_–O), 3.53 ppm (1 H, m, CH_3_–C*H*_2_–O), 1.41 ppm (3 H, d, C*H*_3_–CH–(O)_2_), 1.18 ppm (3 H, t, C*H*_3_–CH_2_–O).

^1^H NMR proCEA (CDCl_3_, 300 MHz) δ: 6.32 ppm (1 H, dd, C*H*_2_=CH–C(O)), 6.06 ppm (1 H, dd, CH_2_=C*H*–C(O)), 5.93 ppm (1 H, q, CH_3_–C*H*–(O)_2_), 5.80 ppm (1 H, dd, C*H*_2_=CH–C(O)), 4.39 ppm (2 H, t, O–C*H*_2_–CH_2_–C(O)), 3.64 ppm (1 H, m, CH_3_–C*H*_2_–O), 3.48 ppm (1 H, m, CH_3_–C*H*_2_–O), 2.66 ppm (2 H, t, O–CH_2_–C*H*_2_–C(O)), 1.35 ppm (3 H, d, C*H*_3_–CH–(O)_2_), 1.14 ppm (3 H, t, C*H*_3_–CH_2_–O).

^13^C NMR proCEA (CDCl_3_, 300 MHz) δ: 170 ppm (O–CH_2_–CH_2_–*C*(O)–O), 166 ppm (CH_2_=CH–*C*(O)–O), 131 ppm (*C*H_2_=CH–C(O)), 128 ppm (CH_2_=*C*H–C(O)), 96 ppm (CH_3_–*C*H–(O)_2_), 64 ppm (CH_3_–*C*H_2_–O), 60 ppm (O–*C*H_2_–CH_2_–C(O)), 34 ppm (O–CH_2_–*C*H_2_–C(O)), 20 ppm (*C*H_3_–CH–(O)_2_), 14 ppm (*C*H_3_–CH_2_–O). 

## 3. Results and Discussion

### 3.1. Synthesis of PBA-b-PDMAEA Diblock Copolymers

**Figure 2:**
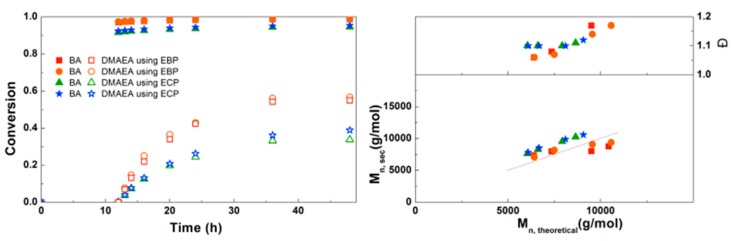
(**Left**): conversion versus time plot for Cu(0)-mediated one-pot block copolymerization of BA and DMAEA using [BA]:[DMAEA]:[EBP]:[Me_6_TREN]:[CuBr_2_] = 50:50:1:0.15:0.1 and [BA]:[DMAEA]:[ECP]:[Me_6_TREN]:[CuBr_2_] = 50:50:1:0.15:0.1, 3.0 M in DMF at 25 °C and 12.5 mm^2^/mL Cu(0) wire. A 3.0 M solution of DMAEA in DMF was added after 12 h reaction time. (**Right**): corresponding molecular weight and dispersity versus theoretical molecular weight plot.

To assess the feasibility of making amphiphilic block copolymers of PBA with PDMAEA and PEEA, Cu(0)-mediated homopolymerizations of DMAEA and EEA were first investigated. These results can be found in the Supplementary Information (Figures S4 and S5), revealing that well-defined homopolymers with *Đ* ~1.2 could be obtained using non-optimized conditions. For the one-pot block copolymerization of BA with DMAEA, the first experiment was performed to determine which initiator is the most suitable for this reaction (Figure 2). The results of the block copolymerizations are shown as conversion versus time plots instead of first order kinetic plots, because the ln([M]_0_/[M]) of the second monomer is generally not linear and, in addition, the conversion of both monomers is clearer in the conversion versus time plots. In these reactions, first a homopolymerization of BA was performed under the previously optimized conditions of [BA]:[initiator]:[Me_6_ TREN]:[CuBr_2_] = 50:1:0.15:0.1 at 3 M monomer concentration in DMF with a reaction volume of 4 mL. After 12 h of reaction time, when BA had reached almost full conversion for all reactions, 4 mL of a 3 M solution of DMAEA was added to the reactors, after which the reactions were monitored by taking samples at different time intervals. Because it was expected that DMAEA may react with the halogen end group of the growing polymer chain, a bromide and a chloride–containing initiator were compared. The experiments in our previous paper showed that EBP was a good initiator for the polymerization of BA [1], and ECP was chosen for its similar structure. In this experiment, CuBr_2_ was used as Cu(II) source for both initiators, to be able to compare only the effect of the initiator itself. Both experiments were performed in duplicate and show good reproducibility. The polymerizations using EBP show higher conversion of both BA and DMAEA than the polymerizations using ECP, due to its higher activation rate [24]. At the last data point, after 48 h of reaction time, almost no increase in conversion was seen anymore. This is likely from a loss of radicals, which may be due to increasing buildup of Cu(II) in the system. As Cu(II) acts as deactivator, an increased amount will slow down the polymerizations. Looking at the SEC data, the experimental molecular weights are in good agreement with *M*_n,th_ for both initiators, with a linear increase of the *M*_n_ with increasing conversion of DMAEA. Importantly, the dispersity of the polymerizations with ECP is constant, while the dispersity of the EBP polymerizations increases with conversion.

**Figure 3:**
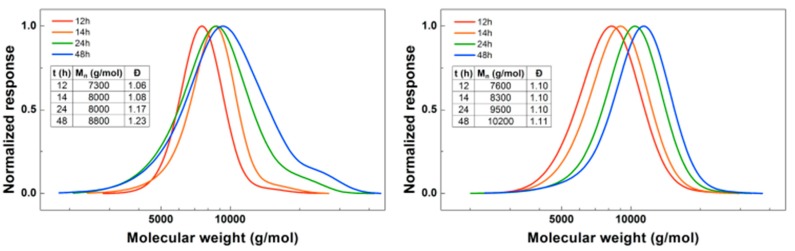
(**Left**): SEC traces for PBA-*b*-PDMAEA using EBP as initiator. (**Right**): SEC traces for PBA-*b*-PDMAEA using ECP as initiator.

When looking in detail at the SEC traces of the polymerizations (Figure 3), the difference between the reactions with the two different initiators becomes more obvious. While the homopolymerization of BA using EBP shows a well-controlled polymerization with a dispersity of 1.06, after addition of DMAEA the polymer peak broadens and the dispersity increases significantly. With ECP as an initiator, the dispersity for PBA is slightly higher at 1.10. However, when DMAEA is added a clean shift of the peak is observed, indicating growth of the polymer, and the dispersity and shape of the peak stay constant. So while ECP is a less effective initiator for BA compared to EBP, it works much better for DMAEA than EBP. This is probably due to the quarternization that can occur between the amine group of DMAEA and the ω-end of the polymer [13]. This is less pronounced when using a chloride-containing initiator compared to a bromide-containing initiator as alkyl bromides are stronger alkylating agents than alkyl chlorides. Therefore, further reactions were performed using ECP and CuCl_2_. DMAEA containing polymers obtained with both initiators show some tailing at low molecular weight, which may be due to the polymers interacting with the columns.

**Figure 4:**
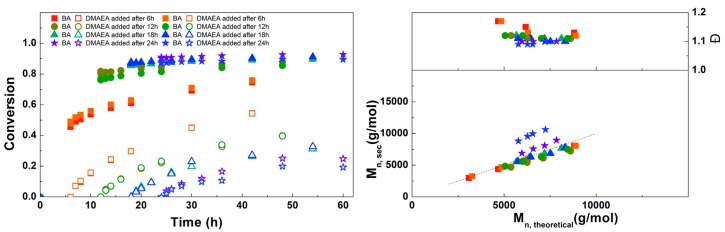
(**Left**): conversion versus time plot for Cu(0)-mediated one-pot block copolymerization of BA and DMAEA using [BA]:[DMAEA]:[ECP]:[Me_6_TREN]:[CuCl_2_] = 50:50:1:0.15:0.1, 3.0 M in DMF at 25 °C and 12.5 mm^2^/mL Cu(0) wire. A 3.0 M solution of DMAEA in DMF was added after 6, 12, 18 or 24 h reaction time. (**Right**): corresponding molecular weight and dispersity versus theoretical molecular weight plot.

For the next experiment, the solution of DMAEA was added to the reaction at different times to find out which would result in the most defined diblock copolymer (Figure 4). When the DMAEA is added after 6 h, BA conversion is still low, so this leads to the formation of a mixed second block. The same is true for 12 h, although to a lesser extent. When DMAEA is added after 18 or 24 h, almost no further conversion of BA is seen after addition of DMAEA, so in these reactions almost pure diblock copolymers are formed. In further reactions, DMAEA was added after 18 h, because in that case the final conversion of DMAEA was a little higher. *M*_n,SEC_ is in good agreement with *M*_n,th_ for all polymerizations, with all dispersities around 1.1, showing good control over the polymerizations.

**Figure 5:**
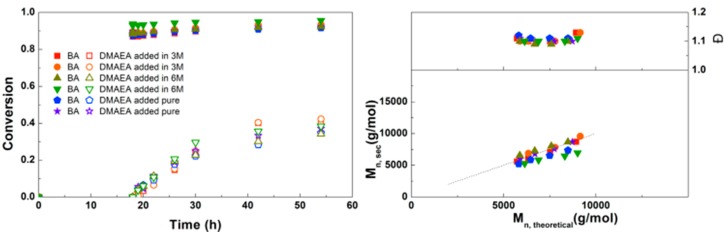
(**Left**): conversion versus time plot for Cu(0)-mediated one-pot block copolymerization of BA and DMAEA using [BA]:[DMAEA]:[ECP]:[Me_6_TREN]:[CuCl_2_] = 50:50:1:0.15:0.1, 3.0 M in DMF at 25 °C and 12.5 mm^2^/mL Cu(0) wire. A 3.0 M or 6.0 M solution of DMAEA in DMF or pure DMAEA was added after 18 h reaction time. (**Right**): corresponding molecular weight and dispersity versus theoretical molecular weight plot.

The addition of DMAEA as pure monomer or as 3 M or 6 M solution in DMF was studied next. It was expected that a higher concentration would lead to a faster polymerization, due to higher availability of the monomer, though an increase in viscosity may also lead to slower polymerization due to diffusion limitations as well as higher dispersity due to inadequate mixing. As shown in Figure 5, no significant difference is observed between the reactions, so the increase in polymerization rate is negligible. Probably the small effects of increasing both concentration and viscosity cancel each other out, so the concentration of DMAEA was kept at 3.0 M for further reactions. As in the previous reactions, good control over molecular weight with a dispersity of 1.1 was obtained. This is lower than the dispersities reported by Whittaker et al. for diblock copolymers of different alkyl acrylates, although different reaction conditions were used and almost full conversion was obtained in multiple iterative steps.[5] More recent papers show dispersities of 1.09 for diblock poly(methyl acrylate) and 1.05 for poly(methyl acrylate-*block*-*tert*-butyl acrylate) [6], while a dispersity of 1.13 was obtained with photoinitiated Cu(0)-mediated synthesis of poly(methyl acrylate-*block*-*tert*-butyl acrylate) [8]. So although a lower final conversion is obtained than in these examples using simple acrylates, the similar dispersity indicates that these polymerizations are similarly well-controlled.

### 3.2. Synthesis of PDMAEA-b-PBA-b-PDMAEA Triblock Copolymers

After successful optimization of the PBA-*b*-PDMAEA diblock copolymer synthesis, a bifunctional chloride-containing initiator was used to synthesize PDMAEA-*b*-PBA-*b*-PDMAEA triblock copolymers. As this initiator is not commercially available, it was synthesized from 2-chloropropionic acid and ethylene glycol using EDC and DMAP. The product was washed with HCl, distilled water and NaHCO_3_ to remove impurities, and as all impurities are water-soluble, column chromatography was not needed. The ^1^H NMR spectrum of this initiator can be found in the Supplementary Information (Figure S7).

**Figure 6:**
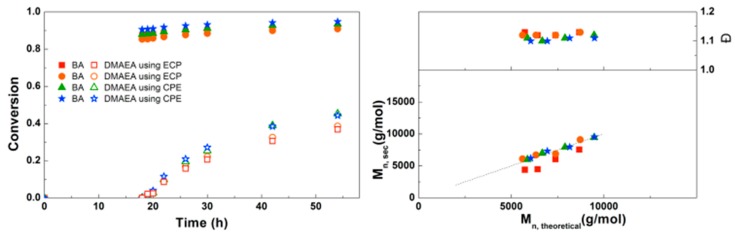
(**Left**): conversion versus time plot for Cu(0)-mediated one-pot block copolymerization of BA and DMAEA using [BA]:[DMAEA]:[ECP]:[Me_6_TREN]:[CuCl_2_] = 50:50:1:0.15:0.1 and [BA]:[DMAEA]:[CPE]:[Me_6_TREN]:[CuCl_2_] = 50:50:1:0.3:0.2, 3.0 M in DMF at 25 °C and 12.5 mm^2^/mL Cu(0) wire. A 3.0 M solution of DMAEA in DMF was added after 18 h reaction time. (**Right**): corresponding molecular weight and dispersity versus theoretical molecular weight plot.

In Figure 6 the polymerizations using the monofunctional ECP and bifunctional CPE are compared. Because CPE can be considered as two initiators, the amount of Me_6_TREN and CuCl_2_ was doubled compared to ECP. Conversions of BA and DMAEA are slightly higher using CPE than with ECP, which can be explained by the lower M/I ratio when CPE is considered as two initiators. Overall the reaction kinetics are very similar between the two initiators. The SEC traces for the triblock copolymerizations can be found in the Supplementary Information (Figure S8). A similar shift in molecular weight is observed as in earlier reactions with the monofunctional initiator (Supplementary Information, Figure S8), indicating that the triblock copolymer chains grow steadily with time and no significant side-reactions occur. 

**Figure 7:**
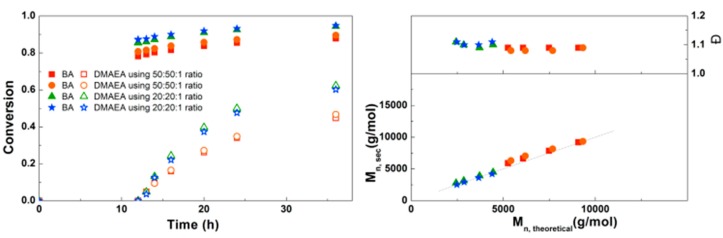
(**Left**): conversion versus time plot for Cu(0)-mediated one-pot block copolymerization of BA and DMAEA using [BA]:[DMAEA]:[CPE]:[Me_6_TREN]:[CuCl_2_] = 50:50:1:0.3:0.2 and [BA]:[DMAEA]:[CPE]:[Me_6_TREN]:[CuCl_2_] = 20:20:1:0.3:0.2, 3.0 M in DMF at 25 °C and 12.5 mm^2^/mL Cu(0) wire. A 3.0 M solution of DMAEA in DMF was added after 12 h reaction time. (**Right**) corresponding molecular weight and dispersity versus theoretical molecular weight plot.

Inspired by the successful synthesis of defined PDMAEA-*b*-PBA-*b*-PDMAEA triblock copolymers, it was attempted to prepare triblock copolymers with different lengths (Figure 7). Here it is clear that the polymerization rate of both the BA and the DMAEA is lower when a higher monomer to initiator ratio is used, as may be expected from the lower initiator and ligand concentrations. In all samples a good agreement of *M*_n,SEC_ with *M*_n,th_ and a dispersity around 1.1 was found, indicating that the triblock copolymerizations are well-controlled. This triblock copolymerization was also performed on a larger scale using schlenk techniques leading to 8 g of polymer with similar M_n_, composition and Đ, which can be found in the Supplementary Information, Table S1.

### 3.3. Synthesis of PBA-b-PEEA Diblock Copolymers

**Figure 8:**
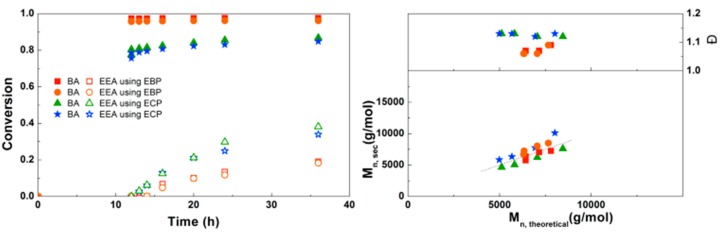
(**Left**): conversion versus time plot for Cu(0)-mediated one-pot block copolymerization of BA and EEA using [BA]:[EEA]:[EBP]:[Me_6_TREN]:[CuBr_2_] = 50:50:1:0.15:0.1 and [BA]:[EEA]:[ECP]:[Me_6_TREN]:[CuCl_2_] = 50:50:1:0.15:0.1, 3.0 M in DMF at 25 °C and 12.5 mm^2^/mL Cu(0) wire. A 3.0 M solution of EEA in DMF was added after 12 h reaction time. (**Right**): corresponding molecular weight and dispersity versus theoretical molecular weight plot.

For the block copolymerizations of BA and EEA, again a first experiment was performed to compare EBP and ECP as initiators. As shown in Figure 8, using EBP gives almost full conversion of BA after 12 h, while the final conversion of EEA is relatively low (20%). Using ECP as initiator, BA does not reach full conversion but the EEA reaches higher conversion compared to EBP. This can be explained by the faster BA polymerization leading to more buildup of Cu(II), eventually leading to a slower polymerization in the later stages of the reaction as the diblock copolymer propagates. For both initiators the *M*_n_ is close to *M*_n,th_, but dispersities are slightly higher when ECP is used, as expected due to slower initiation.

When the SEC traces of these polymerizations are compared (Supplementary Information, Figure S9), it is clear that the polymerizations using ECP show significantly broader peaks, indicative for the slower initiation. With EBP, some tailing at the low *M*_n_ side leads to a small increase in dispersity at higher conversion, possibly due to the presence of dead PBA chains resulting from too high conversion in the first step. Nonetheless, EBP was chosen as the most suitable initiator for the synthesis of block copolymers.

**Figure 9:**
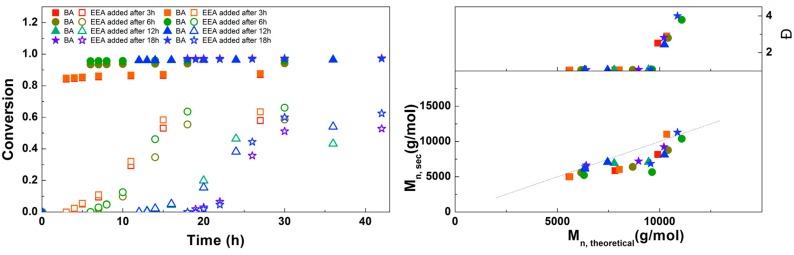
(**Left**): conversion versus time plot for Cu(0)-mediated one-pot block copolymerization of BA and EEA using [BA]:[EEA]:[EBP]:[Me_6_TREN]:[CuBr_2_] = 50:50:1:0.15:0.1, 3.0 M in DMF at 25 °C and 12.5 mm^2^/mL Cu(0) wire. A 3.0 M solution of EEA in DMF was added after 3, 6, 12 or 18 h reaction time (closed symbols: BA, open symbols: EEA). (**Right**): corresponding molecular weight and dispersity versus theoretical molecular weight plot.

To optimize the polymerizations with EBP, an experiment was performed in which the solution of EEA was added at different times during the reaction (Figure 9). After 3 h the conversion of BA is not yet complete, but when EEA is added after 6 h or later almost no further conversion of BA is seen. Final conversions of EEA in this experiment are slightly higher than in some of the other experiments, which may be due to freshness of the used chemicals, as both Me_6_TREN and EEA were distilled immediately prior to this experiment. In all these reactions the conversion of EEA stops around 12 h after the monomer is added. The SEC results generally show a good agreement of *M*_n,SEC_ with *M*_n,th_. Dispersities are around 1.05 at lower conversion, but above around 50% conversion of EEA a large increase in dispersity is seen, together with the formation of a second peak at high *M*_n_. This is believed to be caused by crosslinking through the formation of anhydrides, which was reported earlier for this monomer.[17,25] Unfortunately, we were not able to prevent this during this reaction. However, the crosslinks can be broken through hydrolysis of the anhydrides, which could be achieved by simply stirring the polymers in water (see Supplementary Information, Figure S10 for details). Based on these results, the optimal addition time of EEA was chosen at 6 h.

### 3.4. Synthesis of PEEA-b-PBA-b-PEEA Triblock Copolymers

**Figure 10:**
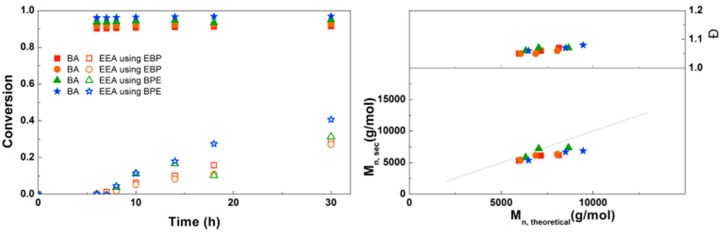
(**Left**): conversion versus time plot for Cu(0)-mediated one-pot block copolymerization of BA and EEA using [BA]:[EEA]:[EBP]:[Me_6_TREN]:[CuBr_2_] = 50:50:1:0.15:0.1 and [BA]:[EEA]:[BPE]:[Me_6_TREN]:[CuBr_2_] = 50:50:1:0.3:0.2, 3.0 M in DMF at 25 °C and 12.5 mm^2^/mL Cu(0) wire. A 3.0 M solution of EEA in DMF was added after 6 h reaction time. (**Right**): corresponding molecular weight and dispersity versus theoretical molecular weight plot.

A bifunctional bromine-containing initiator, BPE, was used for the synthesis of PEEA-*b*-PBA-*b*-PEEA triblock copolymers using the optimal conditions (Figure 10). Similar results were obtained for polymerizations with a monofunctional or bifunctional initiator, which was also seen for PDMAEA-*b*-PBA-*b*-PDMAEA triblock copolymers. Dispersities are below 1.1 in all reactions confirming well-controlled polymerizations leading to defined triblock copolymers. No crosslinking due to anhydride formation was observed in these reactions. This triblock copolymerization was also performed on a larger scale using schlenk techniques with different block sizes of EEA, leading to 8 g of defined triblock copolymer for each reaction (Supplementary Information, Table S1).

### 3.5. Synthesis of PBA-b-PproCEA Diblock and PproCEA-b-PBA-b-PproCEA Triblock Copolymers

In addition to EEA, proCEA was used as an alternative acidic monomer containing a protective group that could be removed under mild conditions. The synthesis of this new monomer was performed similarly to the previously reported synthesis of EEA and related monomers by acid catalyzed addition of ethyl vinyl ether to CEA [16,18]. To prove the successful synthesis of proCEA, both 1D and 2D NMR spectra were measured, which can be found in the Supplementary Information, Figures S11–S14.

**Figure 11:**
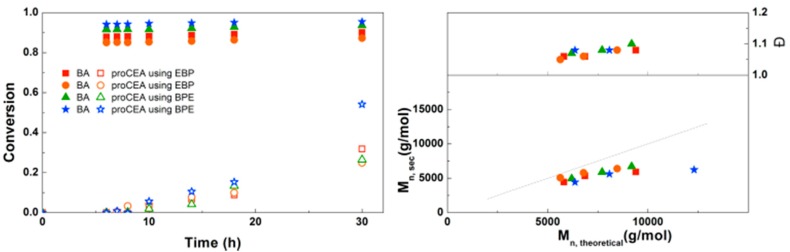
(**Left**): conversion versus time plot for Cu(0)-mediated one-pot block copolymerization of BA and proCEA using [BA]:[proCEA]:[EBP]:[Me_6_TREN]:[CuBr_2_] = 50:50:1:0.15:0.1 and [BA]:[proCEA]:[BPE]:[Me_6_TREN]:[CuBr_2_] = 50:50:1:0.3:0.2, 3.0 M in DMF at 25 °C and 12.5 mm^2^/mL Cu(0) wire. A 3.0 M solution of proCEA in DMF was added after 6 h reaction time. (**Right**): corresponding molecular weight and dispersity versus theoretical molecular weight plot.

For the diblock and triblock copolymerization of BA with proCEA, similar reaction conditions were used as those previously optimized for the block copolymerizations of BA with EEA. Figure 11 shows that the reaction kinetics observed are quite similar to the polymerizations of EEA. One reaction shows a significantly higher conversion at 30 h, which seems to be a faulty data point because SEC shows a much lower M_n_. It is possible that some of the monomer in this particular sample was deprotected due to moisture, leading to an inaccurate GC measurement. Other than that, the polymerizations seem to be well-controlled. This triblock copolymerization was also performed on larger scale using schlenk techniques leading to ~5 g of defined triblock copolymer (Supplementary Information, Table S1).

## 4. Conclusions

The synthesis of amphiphilic diblock and triblock copolymers of BA with DMAEA, EEA and proCEA was optimized via high-throughput Cu(0)-mediated polymerization. Depending on the reaction conditions used, either a mixed monomer second block, containing a small amount of BA, or a near perfect block copolymer was produced. When DMAEA was used as the second monomer, reactions using EBP as initiator showed quarternization, while this was not observed when ECP was used as initiator and block copolymers with a dispersity of 1.1 could be prepared successfully. Although side reactions from the amine group of the DMAEA were expected, under the used conditions these were suppressed sufficiently to yield well-defined polymers.

When using EEA as the second monomer, unintended deprotection of the monomer can lead to disruption of the copper-ligand catalyst and cause uncontrolled polymerization. The formation of anhydrides can also cause problems by crosslinking polymers. However, under the chosen reaction conditions this was largely avoided and defined block copolymers of BA and EEA were synthesized successfully. The newly synthesized monomer proCEA could also be polymerized with good control. The optimal addition time of the second monomer was found to be 18 h for DMAEA, due to the slower polymerization rate when ECP is used as initiator, and 6 h for EEA and proCEA. Using these times, almost full conversion of BA was obtained and pure (tri)block copolymers were produced.

## Supplementary Materials

Supplementary materials are available online at www.mdpi.com/2073-4360/9/8/320/s1. Figure S1: Deprotection of PEEA to PAA. Figure S2: Initiators used in this paper. From left to right: EBP, ECP, BPE and CPE. Figure S3: Synthesis of proCEA. Figure S4: Left: first order kinetic plot for Cu(0)-mediated polymerization of DMAEA using different conditions and 14.3 mm^2^/mL Cu(0) wire. Right: corresponding molecular weight and dispersity vs. conversion plot. Figure S5: Left: first order kinetic plot for Cu(0)-mediated polymerization of EEA using different conditions and 12.8 mm^2^/mL Cu(0) wire. Right: corresponding molecular weight and dispersity vs. conversion plot. Figure S6: Proposed structure of branched PBA-*b*-PDMAEA resulting from quarternization of PDMAEA. Figure S7: ^1^H NMR spectrum of CPE. Figure S8: SEC traces for PDMAEA-*b*-PBA-*b*-PDMAEA prepared using CPE as initiator. Figure S9: Left: SEC traces for PBA-*b*-PEEA using EBP as initiator. Right: SEC traces for PBA-*b*-PEEA using ECP as initiator. Figure S10: SEC traces of PBA-*b*-PEEA before and after precipitation and after stirring in water/THF for a longer time. Figure S11: ^1^H NMR spectrum of proCEA. Figure S12: ^1^H COSY NMR spectrum of proCEA. Figure S13: HSQC NMR spectrum of proCEA. Figure S14: HMBC NMR spectrum of proCEA. Numbers in grey are split peaks of protons and carbons directly attached to each other. Table S1: Details of manual triblock copolymerizations.
