# Supplementary material for: One-Pot Synthesis of Charged Amphiphilic Diblock and Triblock Copolymers Via High-Throughput Cu(0)-Mediated Polymerization
Source: Polymers (Basel). 2017 Jul 30;9(8):320. doi: 10.3390/polym9080320 (PMC6418976; doi:10.3390/polym9080320)
Supplement: Supplementary file 1 [file polymers-09-00320-s001.pdf]

polymers

Supplementary information for:

One-pot synthesis of charged amphiphilic diblock and triblock copolymers via high-throughput Cu(0)-mediated polymerization

Lenny Voorhaar[1,2] and Richard Hoogenboom[1*]

1. Supramolecular Chemistry Group, Department of Organic and Macromolecular Chemistry, Ghent University, Krijgslaan 281 S4, 9000 Ghent, Belgium.
2. SIM vzw, Technologiepark 935, 9052 Zwijnaarde, Belgium.

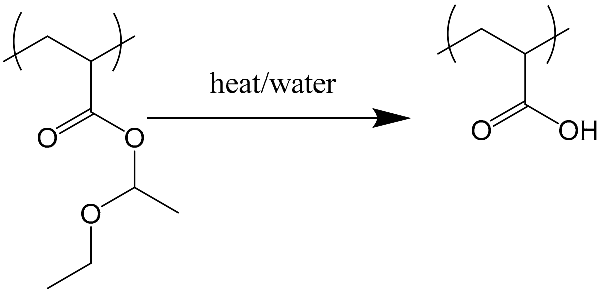

Figure S1. Deprotection of PEEA to PAA.

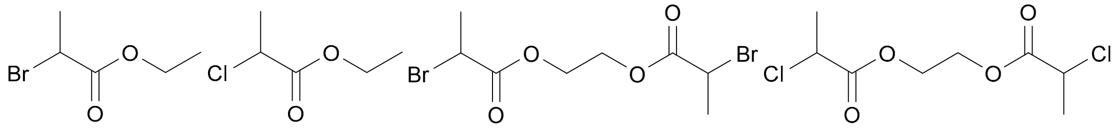

Figure S2. Initiators used in this paper. From left to right: EBP, ECP, BPE and CPE.

Experimental

Synthesis of 1-ethoxyethyl acrylate (EEA) and protected 2-carboxyethyl acrylate (proCEA)

EEA and proCEA were synthesized following a previously published procedure and distilled prior to use.[1] For the synthesis of proCEA (Figure S3), phosphoric acid (109 mg, 1.11 mmol) was weighed into a dry round bottom flask in a glovebox and then taken outside the glovebox, taking care that the phosphoric acid stayed dry. 2-Carboxyethyl acrylate (80 g, 555 mmol) and ethyl vinyl ether (48 g, 666 mmol) were added and the reaction was stirred for two days at room temperature. Hydrotalcite ($Mg_6Al_2(OH)_{16}CO_3{\cdot}4H2O$, ~1 g) was added, stirred for one hour and filtered off. Excess ethyl vinyl ether was removed under reduced pressure and the product was distilled under reduced pressure (80 °C, 1.3 mbar).

Polymers **2017**, *9*, 320; doi:10.3390/polym9080320

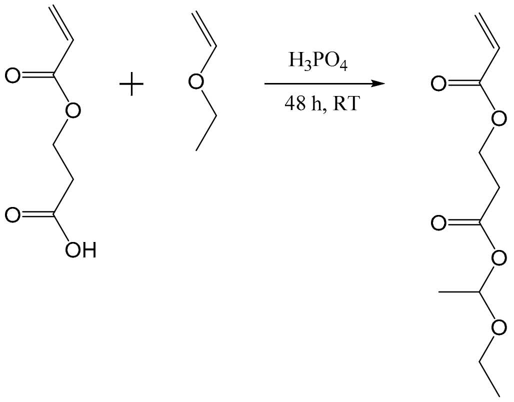

Figure S3. Synthesis of proCEA.

Manual polymerization of PDMAEA-b-PBA-b-PDMAEA

A solution of [BA]:[Me_6TREN]:[$CuCl_2$] = 50:0.3:0.2 at a monomer concentration of 3.0 M in DMF (total volume 21.00 mL) with 12.5 mm^2/mL Cu(0) wire was bubbled with argon for one hour, after which 1 eq. of CPE was added to the reaction mixture with a syringe to start the reaction. The polymerization was run at room temperature for 18.5 hours, after which a degassed solution of DMAEA (3.0 M in DMF, 50 eq.) was added using a cannula. The polymerization was continued to a total reaction time of 44 hours, after which the mixture was diluted with methanol (~10 mL) and stirred with CupriSorb overnight. The mixture was then filtered and a small amount of phenothiazine was added to prevent further polymerization. The solvent was removed under reduced pressure and the resulting mixture was transferred to a glass petridish. The monomer and residual solvent were removed in a vacuum oven overnight.

Manual polymerization of PEEA-b-PBA-b-PEEA

A solution of [BA]:[Me_6TREN]:[$CuBr_2$] = 50:0.3:0.2 at a monomer concentration of 3.0 M in DMF (total volume 21.00 mL) with 12.5 mm^2/mL Cu(0) wire was bubbled with argon for one hour, after which 1 eq. of BPE was added to the reaction mixture with a syringe to start the reaction. The polymerization was run at room temperature for 6 hours, after which a degassed solution of EEA (3.0 M in DMF, 50, 20 and 12 eq. in separate experiments) was added using a cannula. The polymerization was continued to a total reaction time of 48 hours, after which the polymers were precipitated in water, dissolved in THF and dried in a petridish in a vacuum oven.

Manual polymerization of PproCEA-b-PBA-b-PproCEA

A solution of [BA]:[Me_6TREN]:[$CuBr_2$] = 50:0.3:0.2 at a monomer concentration of 3.0 M in DMF (total volume 8.00 mL) with 12.5 mm^2/mL Cu(0) wire was bubbled with argon for one hour, after which 1 eq. of BPE was added to the reaction mixture with a syringe to start the reaction. The polymerization was run at room temperature for 4.5 hours, after which a degassed solution of proCEA (3.0 M in DMF, 25 eq.) was added using a cannula. The polymerization was continued to a total reaction time of 48 hours, after which the mixture was diluted with methanol (~10 mL) and stirred with CupriSorb overnight. The mixture was then filtered, diluted with water and dialyzed (MWCO = 1000) against water and freeze-dried.

Results and discussion

Homopolymerizations of DMAEA and EEA

To assess the feasibility of making amphiphilic block copolymers of PBA with PDMAEA and PEEA, homopolymerizations of DMAEA and EEA were first investigated. The homopolymerization of DMAEA was performed with [DMAEA]:[EBP]:[L]:[$CuBr_2$] = 50:1:0.18:0.05 and 50:1:0.09:0.025 at 3.0 M in DMSO at 25 °C, using both Me_6TREN and PMDETA as ligand. These conditions were chosen based on previous papers that used different monomers.[2] The first order kinetic plot and M_n vs. conversion plot (Figure S4) show that conversions up to 90 % could be obtained in several hours with good control over the M_n. However, the dispersities at high conversion are around 1.2-1.3, which is higher than previously observed for BA homopolymerizations,[3] possibly due to side reactions between the amine of DMAEA and the bromide end-group of the polymer chain, so further optimization is necessary for

Polymers **2017**, *9*, 320; doi:10.3390/polym9080320

the polymerization of DMAEA. The polymerizations using Me6TREN show a higher conversion and lower dispersity than those with PMDETA, which is expected from the higher activation of Me6TREN complexes and was also found for the Cu(0)-mediated polymerization of BA.[3] The reactions with a higher amount of ligand show a higher rate of polymerization, although both ratios give similar dispersities, which is also in line with earlier results.

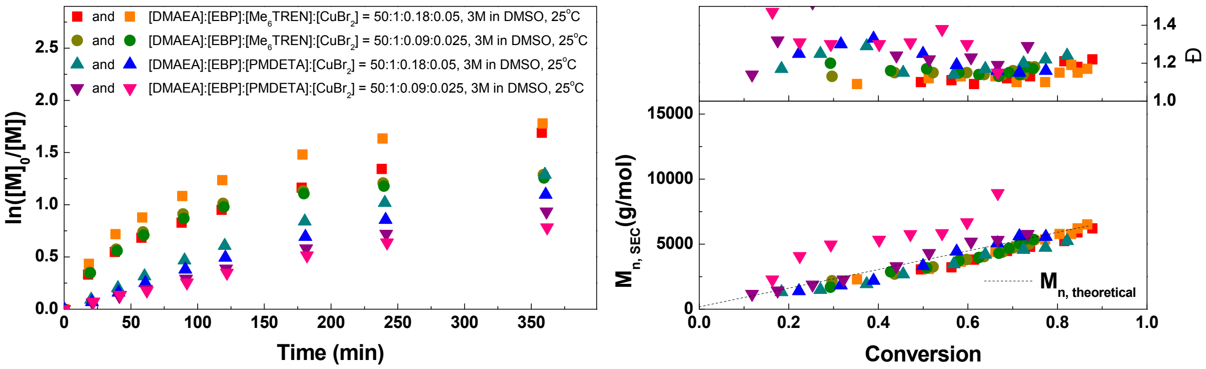

Figure S4. Left: first order kinetic plot for Cu(0)-mediated polymerization of DMAEA using different conditions and 14.3 mm²/mL Cu(0) wire. Right: corresponding molecular weight and dispersity vs. conversion plot.

Homopolymerizations of EEA are shown in Figure S5. Again, reaction conditions from an earlier paper were used as a starting point, with [EEA]:[EBiB]:[Me6TREN]:[CuBr2] = 100:1:0.18:0.05 and lower amount of either ligand or EEA.[2] These reactions were relatively slow, and in some polymerizations an increase in dispersity up to 1.4 is seen, which is quite high for this type of polymerization. This may be from partial deprotection of the EEA to AA, which would interfere with the Me6TREN/copper complex and disrupt the controlled polymerization. This would also explain why the increase in dispersity is seen in only some reactions and not in all duplicates, as the deprotection is difficult to control and is self-catalysing. Similar to the previous experiment, the reactions using a lower ligand ratio are slower. The reactions with a 50:1 ratio of monomer to initiator are faster than those with a 100:1 ratio, even though these were performed at a lower concentration, so it seems that the monomer concentration does not have a very large effect on the rate of polymerization. In summary, more optimization is needed but these preliminary reactions show that DMAEA and EEA can successfully be polymerized using Cu(0)-mediated polymerization. Further optimization was, therefore, directly performed for the block copolymerizations.

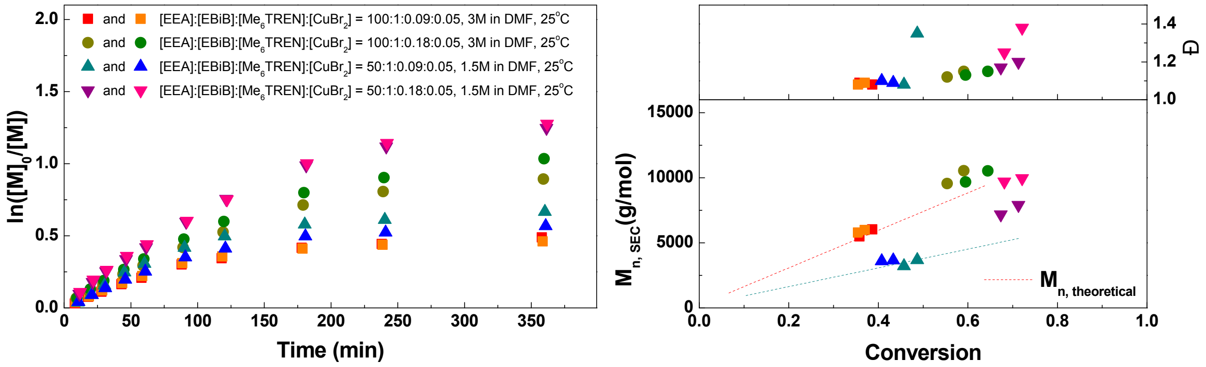

Figure S5. Left: first order kinetic plot for Cu(0)-mediated polymerization of EEA using different conditions and 12.8 mm²/mL Cu(0) wire. Right: corresponding molecular weight and dispersity vs. conversion plot.

Polymers **2017**, *9*, 320; doi:10.3390/polym9080320

polymers

MDPI

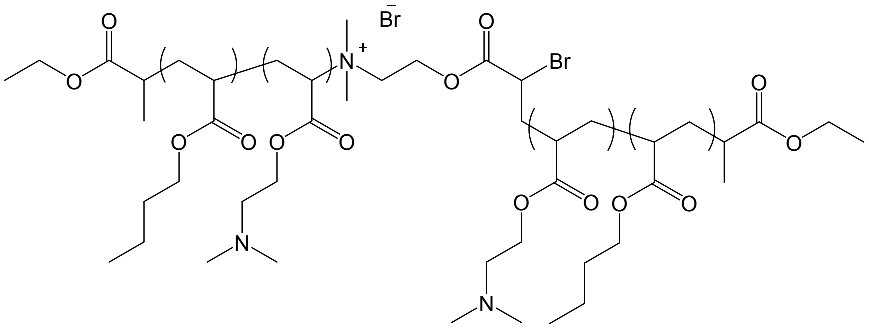

Figure S6. Proposed structure of branched PBA-*b*-PDMAEA resulting from quarternization of PDMAEA.

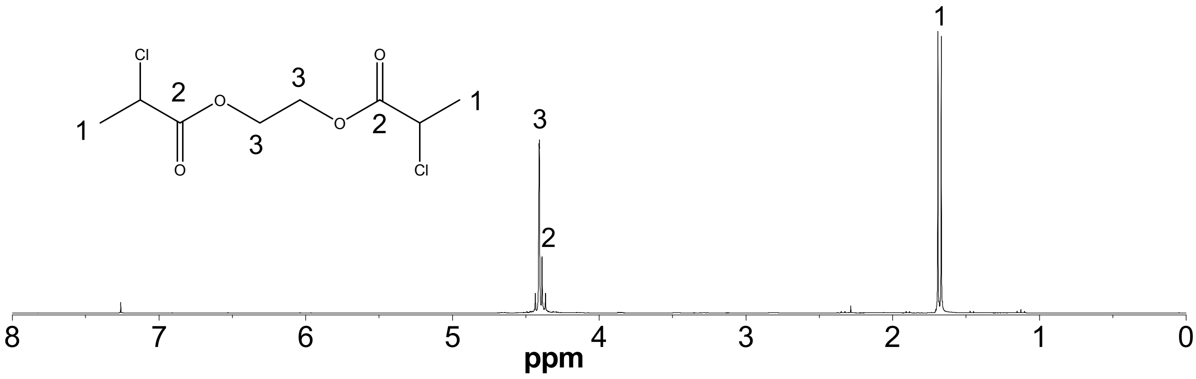

Figure S7. ^{1}H NMR spectrum of CPE.

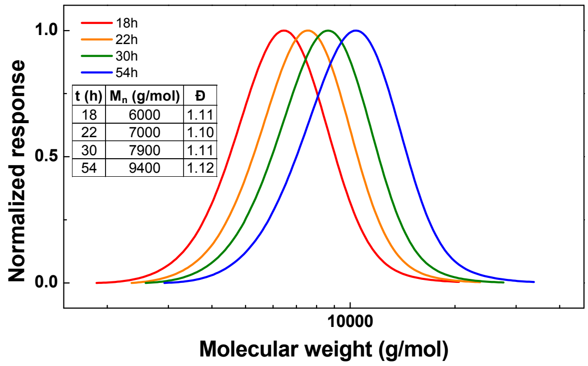

Figure S8. SEC traces for PDMAEA-*b*-PBA-*b*-PDMAEA prepared using CPE as initiator.

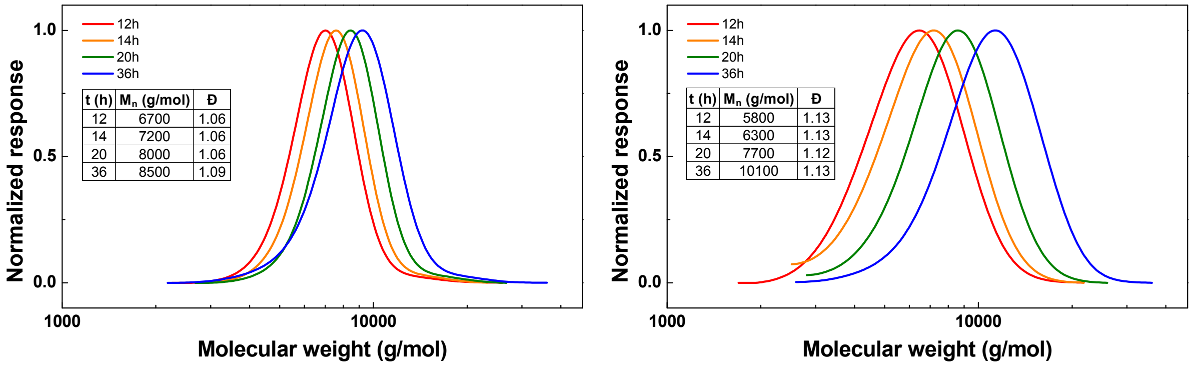

Figure S9. Left: SEC traces for PBA-*b*-PEEA using EBP as initiator. Right: SEC traces for PBA-*b*-PEEA using ECP as initiator.

To prove that the high M_n peaks observed in SEC were indeed caused by the formation of anhydrides between deprotected AA monomers, one of the polymers from this experiment, in which the EEA was added after 6 hours, was precipitated and stirred in a water/THF mixture at room temperature to hydrolyze the anhydrides. The resulting SEC traces are shown in Figure S10. After precipitation in water, the high M_n peak is larger than before precipitation, probably because the larger aggregates of coupled polymer chains precipitate more easily than the individual polymer chains. The size of the high M_n peak decreases with time during stirring in water/THF and eventually it disappears, and only a high M_n shoulder remains, possibly resulting from termination through recombination. Additionally, a small shift of the main polymer peak towards higher molecular weight is observed, which is likely from increased solubility of the acrylic acid groups in the eluent, leading to a larger hydrodynamic radius. The reaction was relatively slow, which is probably due to the polymers being not completely water soluble because of the PBA block. The reaction conditions for this hydrolysis can probably be optimized to complete much faster. Nevertheless, the high M_n peak fully disappeared after several weeks, indirectly demonstrating that the high molar mass polymer fraction could be due to anhydride formation.

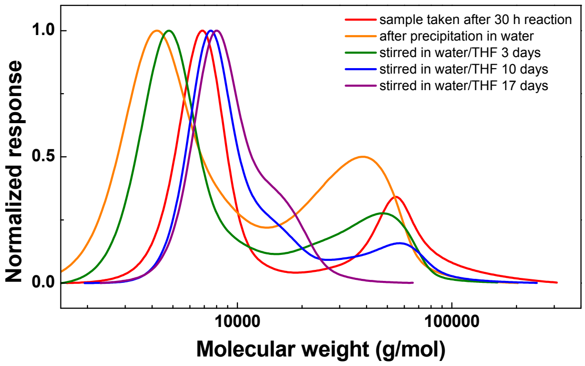

Figure S10. SEC traces of PBA-*b*-PEEA before and after precipitation and after stirring in water/THF for a longer time.

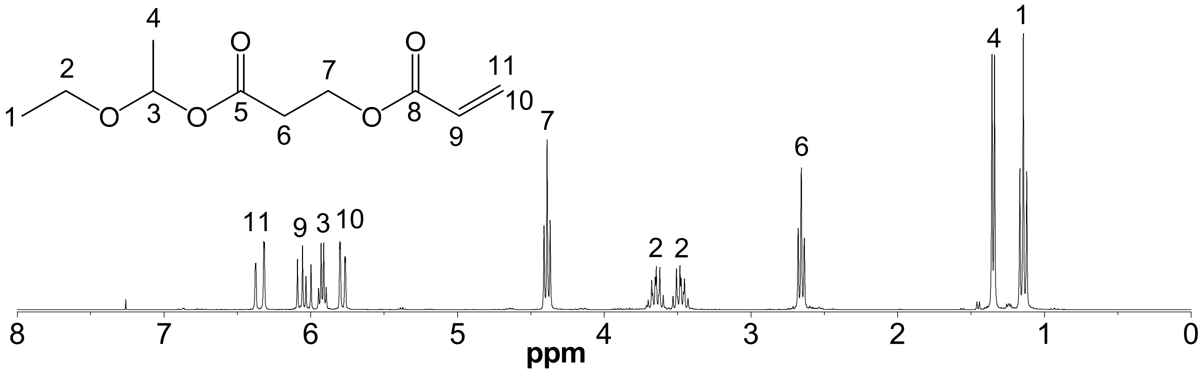

Figure S11. ^{1}H NMR spectrum of proCEA.

Polymers **2017**, *9*, 320; doi:10.3390/polym9080320

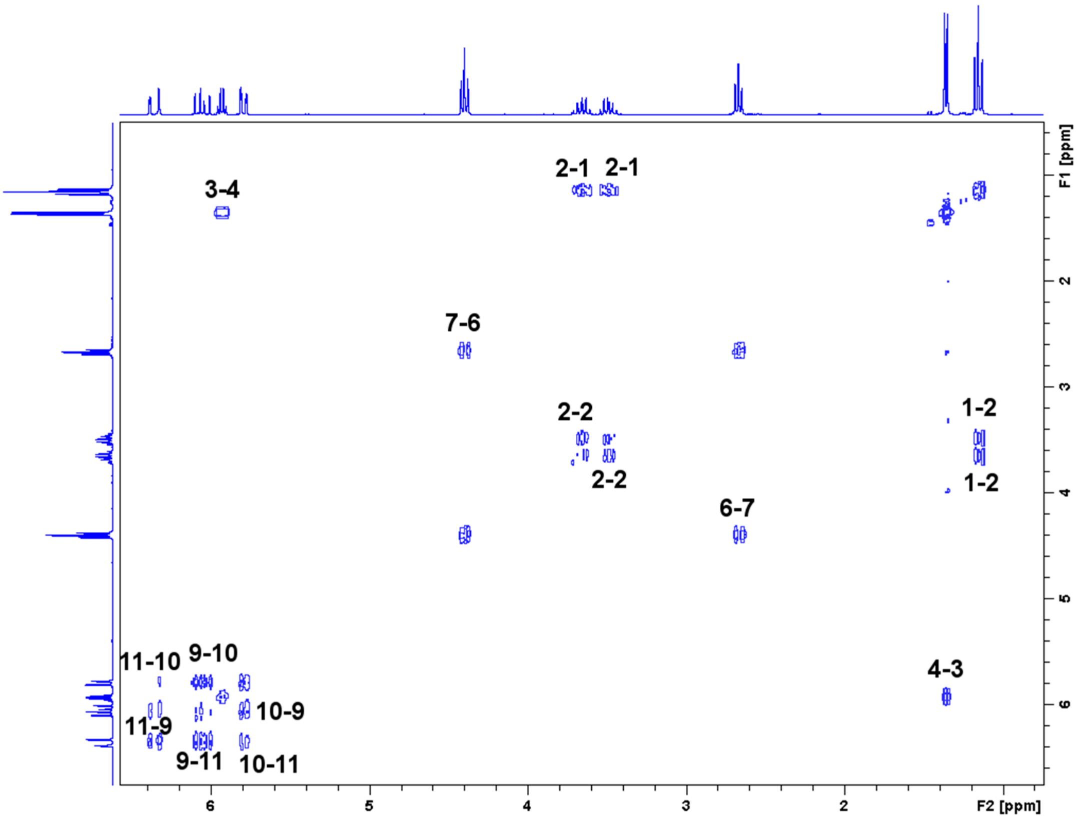

Figure S12. ^{1}H COSY NMR spectrum of proCEA.

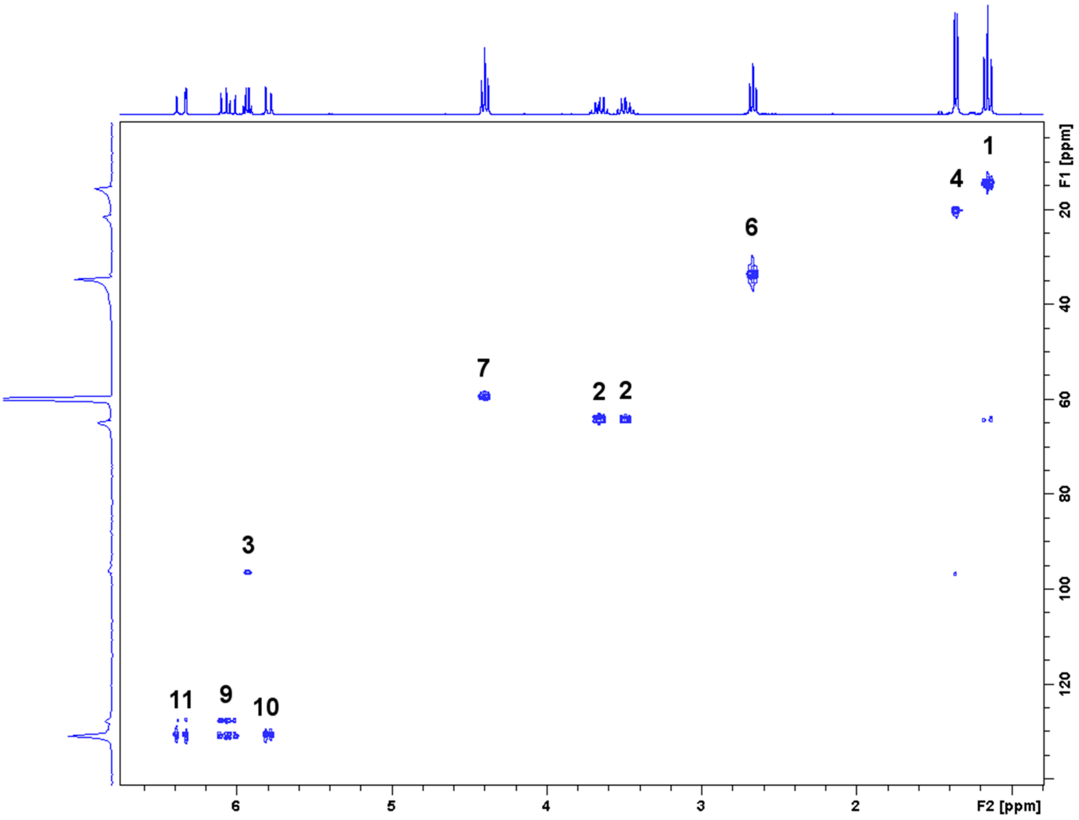

Figure S13. HSQC NMR spectrum of proCEA.

Polymers **2017**, *9*, 320; doi:10.3390/polym9080320

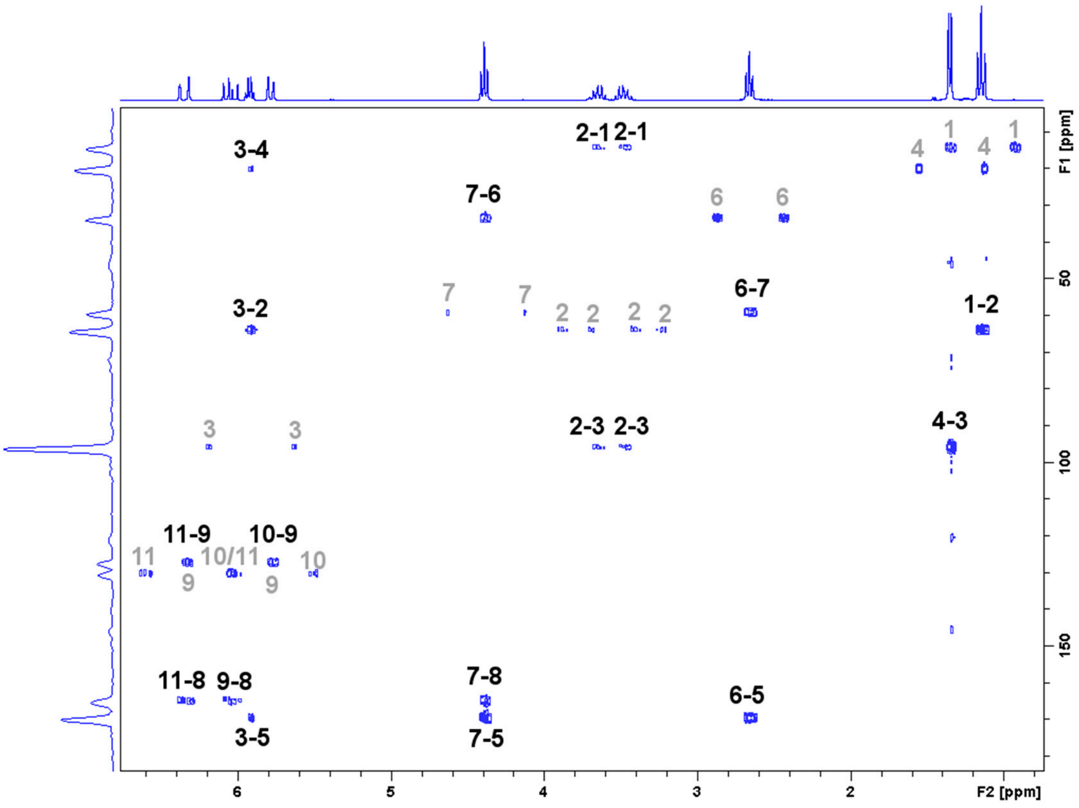

Figure S14. HMBC NMR spectrum of proCEA. Numbers in grey are split peaks of protons and carbons directly attached to each other.

Manual triblock copolymerizations

Triblock copolymerizations under the optimized conditions were manually performed on a larger scale to investigate whether these optimal conditions in the automated synthesizer (8 mL total volume) are also valid on larger scale (~40 mL total volume) (

Polymers **2017**, *9*, 320; doi:10.3390/polym9080320

Table S1). SEC results of each of the polymers before addition of the second monomer and at the end of the polymerization show a clear shift in M_n, towards higher molecular weight as the polymers grow longer. The dispersities observed are slightly higher than seen in the automated polymerizations, which may be due the larger reaction volumes resulting in less efficient stirring and lower temperature control in these exothermic reactions. To test this, it would be possible to perform a larger scale reaction using the automated synthesizer for comparison, but this was not done.

For PproCEA-*b*-PBA-*b*-PproCEA, which is spontaneously deprotected to PCEA-*b*-PBA-*b*-PCEA during the dialysis step of the purification in water, the percentage of BA in the polymer after purification is higher than calculated from monomer conversion, which was measured at 88 %. It seems that the conversion of proCEA is over-estimated by GC, due to monomer degradation during the longer reaction time, and is probably close to 40 %, which would be in line with the results from the automated polymerizations. The exact percentage of AA in the PAA-*b*-PBA-*b*-PAA polymers could not be calculated from NMR, although a difference in viscosity between the polymers after purification could be observed from handling the materials. Full deprotection of EEA, which spontaneously happens during the precipitation step, was confirmed by ^{1}H NMR spectroscopy

Polymers **2017**, *9*, 320; doi:10.3390/polym9080320

Table S1. Details of manual triblock copolymerizations.

polymer	DP of BA (GC)	DP second monomer (GC)	% BA (NMR)	M_n PBA (g/mol, SEC)	Đ PBA (SEC)	M_n triblock (g/mol, SEC)	Đ triblock (SEC)	Yield (g)
PDMAEA-*b*-PBA-*b*-PDMAEA	47	19	68	5700	1.08	7000	1.08	8
PAA-*b*-PBA-*b*-PAA	49	21	-	5900	1.09	7500	1.18	8
PAA-*b*-PBA-*b*-PAA	49	18	-	5800	1.09	6500	1.12	8
PAA-*b*-PBA-*b*-PAA	49	9	-	5900	1.11	6900	1.12	8
PCEA-*b*-PBA-*b*-PCEA	48	21	84	4700	1.11	5300	1.26	5

References

1. Nakane, Y.; Ishidoya, M.; Endo, T. Synthesis and thermal dissociation of polymers having hemiacetal ester moieties, *J. Polym. Sci., Part A: Polym. Chem.*, **1999**, *37*, 609-614.
2. Soeriyadi, A.H.; Boyer, C.; Nystrom, F.; Zetterlund, P.B.; Whittaker, M.R. High-Order Multiblock Copolymers via Iterative Cu(0)-Mediated Radical Polymerizations (SET-LRP): Toward Biological Precision, *J. Am. Chem. Soc.*, **2011**, *133*, 11128-11131.
3. Voorhaar, L.; Wallyn, S.; Du Prez, F.E.; Hoogenboom, R. Cu(0)-mediated polymerization of hydrophobic acrylates using high-throughput experimentation, *Polym. Chem.*, **2014**, *5*, 4268-4276.

Polymers **2017**, *9*, 320; doi:10.3390/polym9080320
